# Hydrocarbon phenotyping of algal species using pyrolysis-gas chromatography mass spectrometry

**DOI:** 10.1186/1472-6750-10-40

**Published:** 2010-05-21

**Authors:** Dinesh K Barupal, Tobias Kind, Shankar L Kothari, Do Yup Lee, Oliver Fiehn

**Affiliations:** 1 Genome Center, University of California, Davis, USA; 2 Department of Botany, University of Rajasthan, Jaipur, India

## Abstract

**Background:**

Biofuels derived from algae biomass and algae lipids might reduce dependence on fossil fuels. Existing analytical techniques need to facilitate rapid characterization of algal species by phenotyping hydrocarbon-related constituents.

**Results:**

In this study, we compared the hydrocarbon rich algae *Botryococcus braunii *against the photoautotrophic model algae *Chlamydomonas reinhardtii *using pyrolysis-gas chromatography quadrupole mass spectrometry (pyGC-MS). Sequences of up to 48 dried samples can be analyzed using pyGC-MS in an automated manner without any sample preparation. Chromatograms of 30-min run times are sufficient to profile pyrolysis products from C8 to C40 carbon chain length. The freely available software tools AMDIS and SpectConnect enables straightforward data processing. In *Botryococcus *samples, we identified fatty acids, vitamins, sterols and fatty acid esters and several long chain hydrocarbons. The algae species *C. reinhardtii, B. braunii *race A and *B. braunii *race B were readily discriminated using their hydrocarbon phenotypes. Substructure annotation and spectral clustering yielded network graphs of similar components for visual overviews of abundant and minor constituents.

**Conclusion:**

Pyrolysis-GC-MS facilitates large scale screening of hydrocarbon phenotypes for comparisons of strain differences in algae or impact of altered growth and nutrient conditions.

## Background

The world requires a sustainable source of energy for the future. Autotrophic organisms have been proposed to reduce the energy dependence of world economy on the fossil oil [1]. Specifically, biofuel derived from microalgae [2] have been under active investigation. Hypothetical yield per hectare, land requirements, eco-friendly production, simple life structure and available scientific technologies are the major advantages to use the microalgae for large-scale biofuel production [3]. The hydrocarbon content of algae, specifically fatty acids, isoprenoids and triacylglycerides [4], have the potential to compensate for future decline of crude oil production [5] if algae growth and harvest can be sustained under economically and energetically feasible parameters. Genetic and environmental factors affect the lipid constituents of microalgae [2,4] as well as algae biomass growth. Depending on the species-strains and conditions, lipids can constituent up to 80% of algal dry mass [2]. For biotechnological applications of algal biofuels [6], existing analytical and computational tools are required to rapidly screen and characterize the strains and environmental conditions. Classic methods such as analysis of total triglycerides use a transmethylation procedure to shave off fatty acids [7] but are not be able to screen for alkanes or uncommon hydrocarbons found in *Botryococcus*. Similarly, current metabolomics methods analyze free fatty acids by trimethylsilylation and GC-TOF mass spectrometry [8] which is not amenable to total fats and pose difficulties to extend towards volatile aliphatics, long chain hydrocarbon and complex lipids in a single analytical method. We here aim at providing a rapid and automated way to assess total hydrocarbon and lipid contents in algae for fast strain discriminations that still can be visualized for the underlying changes in chemical complexity.

Amid the algal species relevant for biofuel research are different strains of *Scenedesmus obliquus*, *Dunaliella salina*, *Botryococcus braunii *and *Chlamydomonas reinhardtii*. One requirement for the use of special strains in biofuel production is that the microalgae produce lipids under normal and stress conditions. Although the triglyceride content of *C. reinhardtii *is very low it is used as a common model organism to study metabolism and metabolic networks under different nutrient and light conditions [9]. Hydrocarbon contents of algal species have been characterized using a variety of analytical tools and procedures. Among the traditional techniques for lipid analysis from algae are gravimetric and spectrophotometric techniques (Nile Red staining) [10]. Gradient centrifugation experiments can be used for rapid analysis and quick comparison of the lipid content of algal species producing high hydrocarbon [11]. More selective and sensitive technologies such as gas chromatography (GC) and liquid chromatography (LC) coupled to mass spectrometric detectors [12] provide quantitative and extensive qualitative data of biofuel constituents of microalgae [13]. Electrospray ionization and atmospheric pressure chemical ionization mass spectrometry [14] and comprehensive two-dimensional liquid chromatography [15] (LCxLC) were used for the analysis of triacylglycerides. Analysis of free fatty acids, sterols and waxes is usually performed with gas chromatographic approaches using flame ionization and mass selective detectors [16]. Plant specific phospholipids and galactolipids can be quantitatively profiled using electrospray ionization tandem mass spectrometry [17]. In addition to these compound-specific techniques, pyrolysis-gas chromatography (pyGC-MS) has been used in combination with pattern recognition for fingerprinting of soils, bacteria, lignin and cellulosic analysis as well as analysis of complex organic matter, but much less applied for lipid profiling. High molecular weight lipids in *B. braunii *were previously characterized using pyGC-MS [18] with the focus on understanding of pyrolysis fragmentations and pitfalls in pyGC-MS, referring to structures that were identified using classical analytical methods such as nuclear magnetic resonance [19] and thin layer chromatography [20]. However, no comprehensive characterization of the overall hydrocarbon patterns was performed using substructure annotations. In addition, these methods had never been shown to be highly useful for rapid and quantitative comparison of multiple species.

We here present a comprehensive approach using existing computational tools and pyrolysis coupled to gas chromatography/mass spectrometry to phenotype the lipid rich *B. braunii *and the model organism *C. reinhardtii *based on their hydrocarbon content. To this end we used mass spectral deconvolution algorithms (AMDIS) [21] which we combined with a result filtering algorithm (SpectConnect) [22] for comparing multiple data sets. In order to get a deeper insight into the chromatogram content we first queried chemical and natural product databases to obtain already known algal compounds. Additionally we performed a substructure analysis [23] of deconvoluted mass spectra together with mass spectral library search. An elution order analysis of several substance classes was performed based on their distinct mass spectral patterns. In order to discriminate between hydrocarbon rich species and hydrocarbon low abundant species we applied unsupervised multivariate statistics and furthermore visualized hydrocarbon abundances with the Cytoscape visualization software. Results can be directly interpreted with respect to the difference in hydrocarbon type and abundance between different algae species that may be important to evaluate strains and hydrocarbon outputs in biofuel reactors.

## Results and discussion

### Pyrolysis GC-MS automatically analyzes multiple samples without any pre-treatment

**Figure 1:**
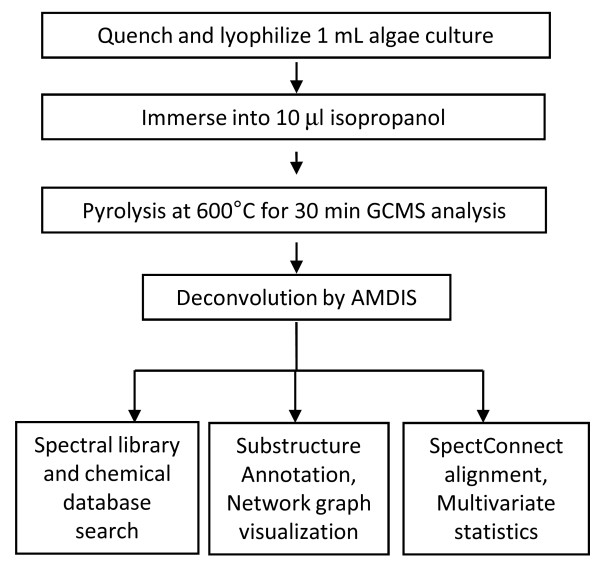
**Flowchart for hydrocarbon phenotyping of algal species by pyrolysis GC-MS**.

**Figure 2:**
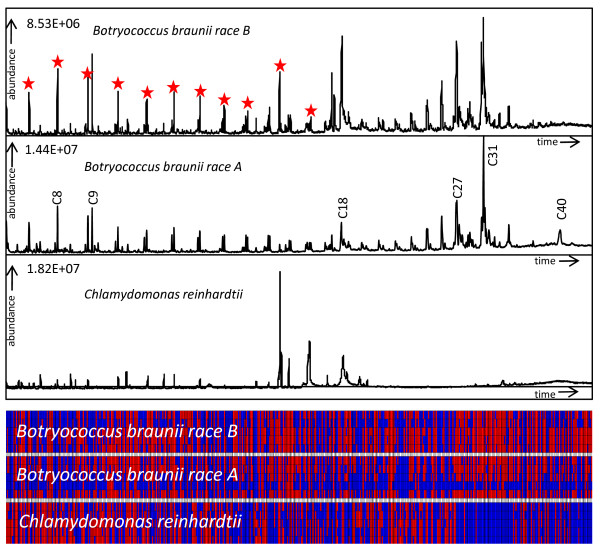
**Algae hydrocarbon chromatograms for extracted ion trace m/z 55**. Stars are indicating structurally similar components that are eluting in a gradual order. Carbon chain lengths are annotated by NIST MS similarity search. The lower pane represents a heatmap of all peaks (in columns) detected by AMDIS after consistency filtering by SpectConnect (red = present, blue = absent. Substructure annotation and identification of algae components are given in supplement S1 and S2.

Thermal pyrolysis of biological macromolecules produces volatile compounds that are easily detected by GC/quadrupole MS for quantitative and qualitative characterization. We have combined pyrolysis GC-MS (pyGC-MS) with mass spectral deconvolution, spectra and structural annotations, consistency filtering and multivariate statistics in order to compare and contrast lipid compositions in algae for biofuel-relevant compounds. The workflow is given in Figure 1, starting with cold quenching of 1 ml-samples of cultures of *B. braunii *race A, *B. braunii *race B and *C. reinhardtii *(around 1 mg dry weight or 10^7 cells, [22]) to stop further metabolic activity. Samples were subsequently freeze-dried and immersed into isopropanol for pyGC-MS analysis, without any extraction step that is known to add to analytical variance. This quantity was enough to produce abundant hydrocarbon pyGC-MS profile data even at split 1:40 injections, with the advantage to use lower split ratios if smaller cell numbers need to be analyzed (Figure 2). Carry-over was tested by pyrolysis of 10 ml of pure isopropanol after every five runs, observing only minor contaminations by algae samples despite the complexity of lipid compositions. The optimum for pyrolysis temperatures was found at 600°C for 12 seconds. Below 600°C we observed lower pyrogram ion intensities, likely to insufficient breakdown and release of lipid constituents. However, above 600°C we found a decline in intact high molecular weight pyrolysis products (Additional file 1, figure S1), indicating an increase in thermal breakdown that would decrease the information content of the pyrograms. The pyrolysis auto-sampler enabled automatic injection of up to 48 lyophilized algae samples into the pyrolysis chamber per analytical sequence. Sequence files can be automatically generated using the study design software SetupX [24] which now enables definition of a variety of technology platforms to store and disseminate metabolome data files. PyGC-MS eliminated the need for labor intensive and time consuming extraction processes in comparison to conventional hydrocarbon analysis of algal species [25,26], hence increasing the sample throughput and lowering the costs for algae analysis. Using the methods detailed above, a total cycle time of 40 minutes was achieved suggesting that pyGC-MS can be utilized for automated studies of 36 samples per day or up to 800 samples per month and instrument, including method blanks and quality control samples. Shorter chromatographic run times of 20 minutes total analysis time (30 min cycle time) resulted in compromised resolution of compounds and cross-contamination in the following samples due to wrap-around of high boiling constituents. If higher throughput would be needed, use of a shorter GC column and a fast flash GC heating option concomitant with increased scan rates would further alleviate data acquisition bottlenecks [27].

### AMDIS and SpectConnect enable straightforward data processing

GC-MS analyses yield complex three-dimensional raw data sets (time *x *mass *x *intensity) which need to be deconvoluted as many fragment ions may be shared between two chromatographically co-eluting compounds. To extract and purify unique mass spectra from complex mixtures, noise analyses and baseline drift corrections are necessary for each ion trace, with subsequent peak picking and mathematical deconvolution using the most unique model ion elution curves [21]. All pyrograms were processed using the freely available AMDIS software [21] using optimized parameters as given in the method section, yielding up to 512 deconvoluted components in *B. braunii*. However, AMDIS is known to evolve a high number of false positive or false negative peak detections, depending on the parameter settings. Therefore, different numbers and identities of components may be detected for biological replicates of the same species. Moreover, when different species or environmental conditions are compared, huge differences in peak numbers and compound identities can be expected. Dot-product similarity alignment algorithms [28-31] will fail in such cases. Instead, we have directly uploaded the *.ELU files resulting from AMDIS processing to the SpectConnect [22] web-tool to find the complement of peaks that are consistently detected in multiple chromatograms of one of the study design classes (e.g. per species). Based on the spectral similarity and retention-time shift corrections, SpectConnect filtered out all peaks that were not detected or below a selected threshold signal, generating three matrices (for relative amount, amount and integrated signals) which were later used for multivariate statistics.

### Hydrocarbons with C8 to C40 carbon chain length were annotated in pyGC-MS pyrograms

Overall, around 500 peaks were consistently detected for the three algae species after SpectConnect filtering, of which about 50% were annotated as hydrocarbons in *Botryococcus *strains but only 33% in *Chlamydomonas *(Table 1). Compounds of carbon chain length from C8 (octane) to C40 (lycopadiene) [32] were observed in the pyGC-MS programs, with increasing retention times from low to high boiling compounds. Aliphatic hydrocarbon profiles in Figure 2 (ion traces m/z 55 as part of the CH_2_-fragmentation series m/z 41, 55, 69, 83) show that not only the number of hydrocarbons but also the absolute hydrocarbon signal abundance was higher in *Botryococcus *strains compared to *Chlamydomonas*. For each aliphatic or aromatic class, structurally similar pyrolysis products (e.g. alkanes or alkenes and branched hydrocarbons) eluted with distinct retention time intervals of around 80 s as validated by serial inspection of their corresponding component mass spectra. Since the chromatograms were processed by AMDIS/SpectConnect, it was feasible to detail absence and presence of peaks in heatmaps (Figure 2). These heatmaps revealed that *C. reinhardtii *indeed did not comprise compounds at more than about 25 carbons, but still yield components between C20-C24 that were not visible in the hydrocarbon ion trace at m/z 55.

**Table 1:** Annotation of pyGC-MS components using AMDIS substructure classifiers.

	Hydrocarbons	Non hydrocarbons	Total components
*C. reinhardtii*	158	318	476
*B. braunii *race A	235	277	512
*B. braunii *race B	258	241	499

AMDIS deconvoluted spectra were subsequently used for compound annotation by searching against the NIST05 mass spectral library at a 70% similarity threshold. Biofuel-related pyrolysis products such as fatty acids and their methyl esters, isoprenoids, sterols, vitamins, aromatics and branched and unbranched alkane-type compounds were identified as intact molecules up to 600°C thermal pyrolysis temperatures (Additional file 2, table S1). Hence, it is likely that components detected by pyGC/MS consist of intact compounds as well as fragments of thermally degraded larger biomolecules. Increasing lengths of side chains were distinguished by an increase of 14 mass units reflecting CH_2_-units. Further peaks were identified using the apparent molecular weights and querying the Dictionary of Natural Products database (DNP). DNP queries retrieved 85 entries for *Botryococcus *DNP of which 17 compounds were matched to pyrograms by molecular weight and mass fragmentation pattern (Additional file 3, table S2). *Botryococcus *is well known for its high content of long chain hydrocarbon contents. Up to 70% of the dry weight are long chain hydrocarbons [32]. Botryococcenes are important hydrocarbons in this alga for which we identified lycopadiene (C40) and botryococcene (C32) in pyGC-MS data based on the molecular ions, mass spectral patterns and retention times (Additional file 3, table S2). The biomarker compound lycopadiene [33] is specifically only found in *B. braunii race A*. DNP comprised only five *Chlamydomonas *metabolites which did not yield additional pyrogram compound annotations.

### Algae components in pyGC-MS data sets can be characterized by substructure networks

**Figure 3:**
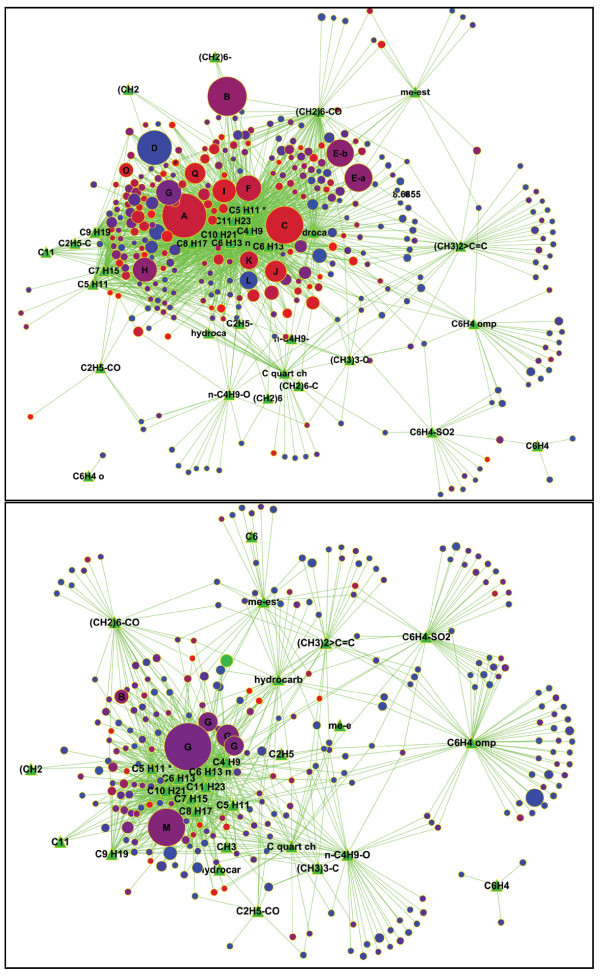
**Substructure annotation network of *Botryococcus braunii *(upper panel) and *Chlamydomonas reinhardtii *(lower panel)**. Nodes represent unique pyrogram hydrocarbons that were clustered by substructure similarities. Node sizes were proportional to the relative component abundances. Color coding was applied by increasing retention time, from blue (low-boiling compounds) to red (high-boiling point compounds). Identified nodes were labeled as A nonacosadiene, B stearic acid, C diepoxyhexadecane, D nonanal, E-a C18 methyl ester1, E-b C18 methyl ester 2, F eicosadiene, G phytadiene, H phytol, I hentriacontadiene, J C30 botryococcene, K eicosadiene, L 1-decane, M palmitic acid, O sterol1, P sterol2, Q epoxynonacosane, R heptacosadiene, S 1-undecane, T 1-nonene.

While tables of identified or annotated compounds detected in pyGC-MS data sets are certainly useful, such tables do not list all detected peaks and are difficult to compare between species or culture conditions with respect to both identity and quantity. More specifically, unidentified components may comprise substantial amounts of the total dry mass and should therefore be related to known compounds and displayed in quantitative terms to obtain visual overview of differences between algal species or strains. We have here applied substructure characterization using automatic analysis of mass spectral patterns using the AMDIS 'Substructure classifier' post processing feature (see workflow Figure 1). The Varmuza substructure classifier algorithm [23] in AMDIS uses various features from mass spectra and annotates them by probable substructures ordered by probability scores, such as 'alkyl hydrocarbon' or 'aromatic naphthalene ring system'. Out of the total list of 150 classifiers (available from the AMDIS online manual at http://www.nist.gov) we selected 26 hydrocarbon related classifiers. In addition, using spectral similarity post processing feature, six different similarity classes were detected. Four of these classes reflected hydrocarbon spectra. A direct comparison found that substructure annotations performed by the Varmuza classifiers in AMDIS yielded results that were highly consistent to the compound identification by NIST MS library search. As many of the compounds belonged to the same chemical class, it can be assumed that mass spectrometry response factors were similar, and hence, that the signal intensities can directly be used to compare the abundance of the different species in hydrocarbon classes. We have visualized the overall outcome of the average qualitative and quantitative presence of algae hydrocarbons in Cytoscape [34] networks (Figure 3 and Additional file 4, figure S2). The abundances and retention time of the components were represented by the circular node sizes and node colors, respectively. Nodes were clustered based on presence in substructure classifiers, represented and labeled as triangular green nodes. Components that shared multiple substructures were structurally similar and clustered more closely together in the network than less related structures. These networks facilitate a quick overview of differences in hydrocarbon contents in species. For example, the node 'M' (palmitic acid) was much higher in *C. reinhardtii *algae than in *Botryococcus*, whereas 'B' (stearic acid) was much more prevalent in *Botryococcus *than in *Chlamydomonas*. This finding indicates that the extra-chloroplastidic elongation of palmitate to stearate is much less active in *Chlamydomonas*, which also does not exert high activity of the biosynthetic pathways for long-chain biosynthesis of unsaturated alkanes (identified compounds A, C, I, F, Q). Conversely, phytadienes (G, the dienes corresponding to the alcohol phytol) are much more abundant in *Chlamydomonas*, pointing to a much more active photosynthetic apparatus that ultimately enables faster growth rates than in *Botryococcus*. Overall, the networks represent multiple features in a single view including peak identifications and classifications, peak abundances and boiling points. The hydrocarbon rich *Botryococcus *algae show a higher number of large red spheres, representing a larger amount of high boiling hydrocarbon compounds. The visual appearance of network instantly reflects the relevant hydrocarbon composition and assists in comparing multiple species and their hydrocarbon contents. Until recently such visualizations were only utilized for large genomic datasets but will play an important role for interpreting multidimensional analytical chemistry related studies.

### Algal species and strains can be easily discriminated by multivariate statistics of pyGC-MS data

**Figure 4:**
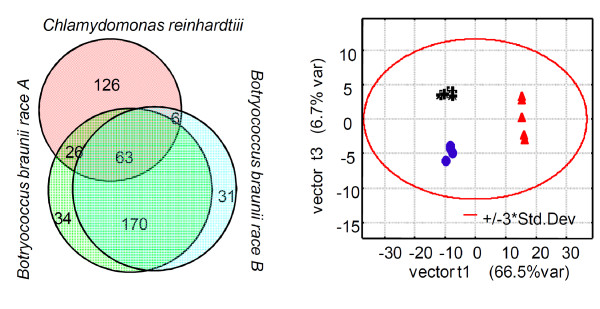
**Species discrimination using pyGC-MS**. Left panel: Venn diagram showing the compound overlap between three algal strains. Right panel: Multivariate principal component analysis using the SpectConnect output matrix of intensities of detected components. Vector 1 discriminates *Chlamydomonas *(red) from *Botryococcus *strains, explaining most of the variance in the data set. Vector 3 comprises only 6.7% of the total variance but clearly distinguishes the *Botryococcus braunii *race A (blue) from race B samples (black).

When comparing the number and overlap of detected compounds, the *Botryococcus *races were apparently highly similar and much more related to each other than to *C. reinhardtii*. The Venn diagram [35] (Figure 4, left panel) showed that 70% of all hydrocarbons were identical between both *Botryococcus *races, whereas *Chlamydomonas *shared only 43% of its hydrocarbons with either of the *Botryococcus *races. One would thus assume that this high overlap in metabolite identity between the *Botryococcus *races would also be reflected in overall quantitative similarities. The quantitative matrices obtained from AMDIS/SpectConnect deconvolution were directly used to display variance between individual replicate culture samples in comparison to variance between the cultures. Unsupervised principal component analysis (PCA, Figure 4 right panel) showed distinct clusters of the three algal species in a two dimensional projection of the scores of first two principal components. Variability within each culture was much lower than between the classes, suggesting a high reproducibility of the pyGC-MS analysis for multiple samples. These results confirm that pyGC-MS with AMDIS/SpectConnect data processing can not only easily distinguish lipid phenotypes of different species but also differentiate pyrograms of closely related races. We therefore suggest that this approach may be extendable to large scale studies involving routine analysis of algae cultures, for example in biofuel technology.

## Conclusions

We have established that pyGC-MS offers a fast track to phenotype algae strains in a cost effective manner, providing both qualitatively and quantitatively important information. Preparation for analysis was minimal and could potentially further robotized, unlike methods that are classically used in lipid analysis or metabolomics. Specifically, constraining AMDIS deconvolution results was important to yield clean and consistent data sets that could be used to compare and contrast hydrocarbon contents. For the first time we have used substructure annotation algorithms, mass spectral library matching and chemical database search to automatically assess hundreds of pyrolysis peaks in algae research. Visualization of result data sets in network graphs offers an improved tool to highlight differences between algae cultures that can much easier be interpreted than commonly used table representations or box-whisker graphs. We suggest that the integrated approach presented here is an efficient strategy for hydrocarbon phenotyping of microalgae in a rapid and automated manner. This method can be applied to small scale as well as large scale research projects considering screening and physiological studies on microalgae for biofuel applications.

## Methods

### Algal Growth and Sampling

Algal samples were harvested and quenched similar to two previously published reports [9,36]. Three different algal strains, *B. braunii *UTEX LB-572, *B. braunii *UTEX-572 and *C. reinhardtii *strain CC125 were used in this study. *Botryococcus *and *Chlamydomonas *strains were cultivated in modified CHU-13 [37,38] and Tris acetate phosphate (TAP) medium respectively at 23°C under constant illumination with cool-white fluorescent bulbs at a fluence rate of 70 μmol m^-2 ^s^-1 ^and with continuous shaking. At the incubation site, 1 mL cell suspensions were injected into 1 mL of -70°C cold quenching solution composed of 70% methanol in water using a thermo block above dry ice. Centrifuge tubes containing the solution during harvest were cooled in a pre-chilled cooling box to keep sample temperature below -20°C. Cells were collected after centrifugation with a rotational speed of 13,200 rpm for 2 min with the centrifuge and rotor cooled at -20°C. Supernatant was decanted and residual liquid carefully removed. The pellet was flash frozen in liquid nitrogen and lyophilized at -50°C in a 2 mL round bottom Eppendorf tube.

### Pyrolysis GC-MS Conditions

Lyophilized samples of *B. braunii *and *Chlamydomonas *were immersed in 2 ml Eppendorf tube with 20 μl of isopropanol. 15 μl of sample were dispensed in pyrolyzer cup and placed on the pyrolysis auto sampler. Pyrolysis was performed with a single-shot pyrolyzer (Model 2020, Frontier Laboratories) directly connected to an Agilent 6890 GC/MS system equipped with a fused silica capillary column (DB5-HT 30 m length, 250 μm inner diameter, 0.25 μm film thickness). An Agilent 5973 quadrupole mass spectrometer was used as detector (EI + at 70 eV). The analysis was performed at a pyrolysis temperature of 600°C for 10 seconds. The pyrolyzer-GC injector interface temperature was set to 320°C. The GC-MS conditions were as follows: oven temperature was held at 50°C for 1 min and then increased up to 325°C at 10°C min^-1 ^and hold at 325°C for 2 min. The GC-MS interface temperature was set to 250°C. Helium was used as a carrier gas with a constant flow of 1.2 ml min^-1^. Split ration was 1:40. The selected mass range was 35-600 Da and the selected scan speed was 2 scans per second. Select pyrograms can be downloaded from the supplement section.

### Computational Methods

The data from GC-MS analysis were deconvoluted in batch mode using the freely available Automated Mass Spectral Deconvolution and Identification System (AMDIS) spectral deconvolution software package (v2.65, NIST Gaithersburg) [21]. AMDIS deconvolution settings were as follow: resolution was medium, sensitivity was low, shape requirement was medium and component width was kept at 10. The SpectConnect online service [39] was used to cross-reference multiple chromatograms for filtering out inconsistent signals [22]. The AMDIS substructure algorithm from the post-processing analysis task was utilized to annotate deconvoluted components with putative substructures. Selected ion chromatograms (SIC) were extracted for 55 m/z using AMDIS. Each component of a given *Botryococcus *sample was searched against the NIST electron impact mass spectral library (NIST05) for compound annotations. AMDIS generated *.FIN files were parsed using Textpad to extract the NIST MS library hits, substructure classifiers, amount, relative abundances and retention time. The following AMDIS substructure classifiers were summarized as hydrocarbon-related compounds: n-C4H9-; n-C4H9-O; n-C4H9-O; C4 H9; C5 H11; C6 H13; C6 H13 n; C7 H15; C8 H17; C9 H19; C10 H21; C11; C11 H23; C11 H23; (CH3)3-C; (CH2); (CH2)6; (CH2)6-; (CH2)6-C; (CH2)6-CO; (CH2)6-CO; (CH3)2 > C = C; (CH3)3-C.

The relationships between components and substructure classifiers were converted into Cytoscape SIF networks (see supplement data). The network was visualized in organic layout using Cytoscape version 2.6. For visualization the node size was adjusted according to the relative abundance of components. Dictionary of Natural Products (DNP 17.1 Copyright 2008 Taylor & Francis Group) [40] was queried for *Botryococcus *and the 85 retrieved compounds were searched in the pyrolysis GC-MS data using the molecular weight as particular m/z to extract the SIC. SpectConnect generated matrices were normalized to the total signal and utilized for principal components analysis in Statistica 8.0 (StatSoft, Tulsa, USA).

## Authors' contributions

DKB and DYL cultured and extracted the *Botryococcus *and *Chlamydomonas *algae. DKB acquired data. DKB and TK performed computational and substructure analyses. SLK helped with experimental designs. DKB, TK and OF wrote the manuscript. All authors have read and approved the manuscript.

## Supplementary Material

Additional file 1 **Figure S1: Effect of temperature on the number of hydrocarbon related pyrolysis products in *Botryococcus braunii*.** -C11H13 substructure shown as representative hydrocarbon. Click here for file

Additional file 2 **Table S1: Identification of pyrolysis products in *Botryococcus braunii *race B using GCMS and NISTMS Library 2005.** Only deconovoluted components are shown here that are annotated as hydrocarbon substructure and that showed more than 70% similarity during NIST05 library searches. Click here for file

Additional file 3 Table S2: Compounds annotations in pyGC-MS based on matching molecular masses and fragment spectra using the Dictionary of Natural Products database. Click here for file

Additional file 4 **Figure S2: Visualization of substructure annotation of using network graph in Cytoscape. Colored spheres are hydrocarbon related components.** Colored triangles are non-hydrocarbon related components. Colors reflect retention time: blue = early elution; red = late elution. Yellow spheres were annotated by hydrocarbon substructure classifiers. Yellow triangles were annotated as non-hydrocarbon substructure classifiers. Click here for file
