# Supplementary figures and images for: Hydrocarbon phenotyping of algal species using pyrolysis-gas chromatography mass spectrometry
Source: BMC Biotechnol. 2010 May 21;10:40. doi: 10.1186/1472-6750-10-40 (PMC2883956; doi:10.1186/1472-6750-10-40)

## Slide 1
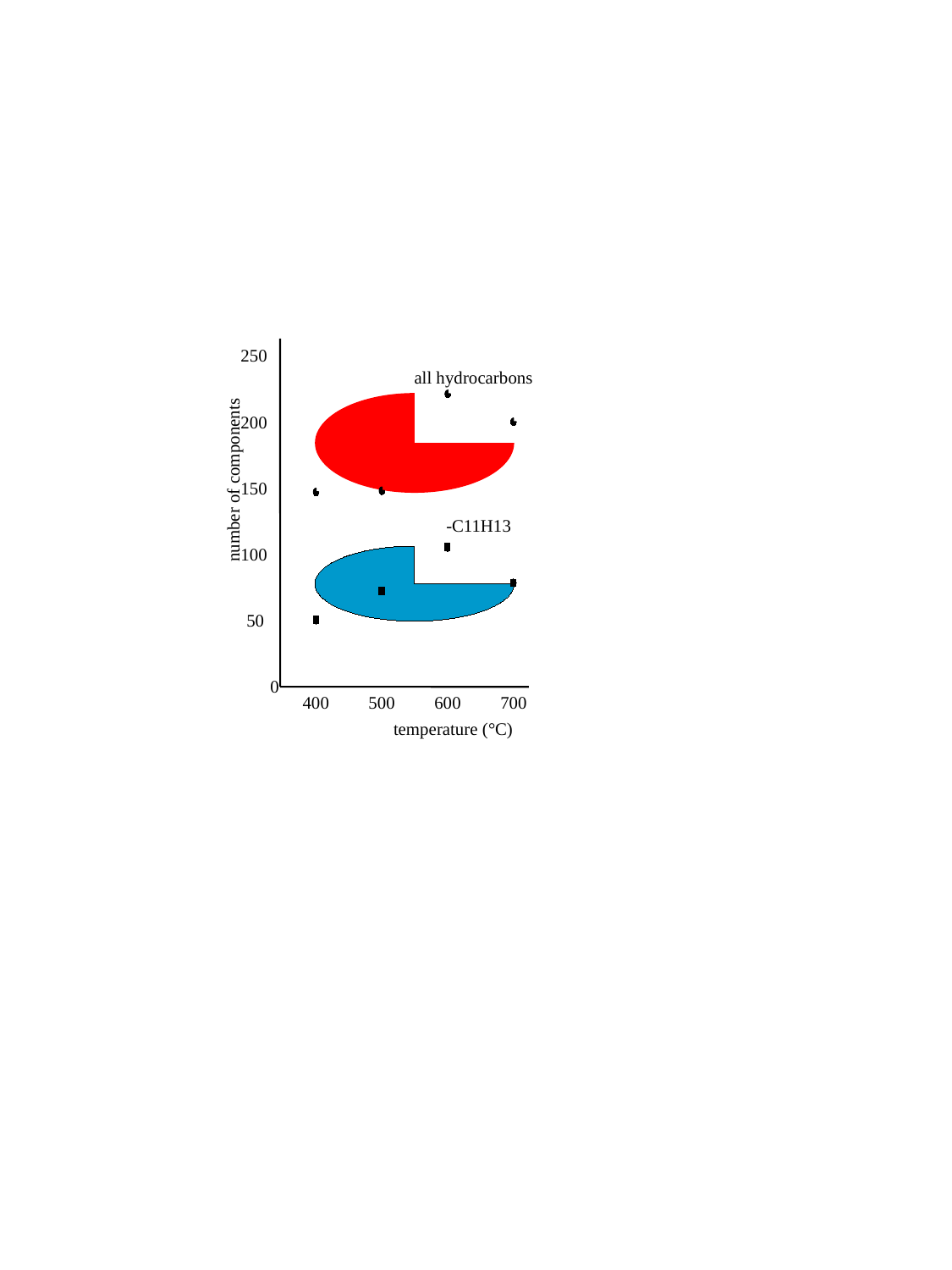

250
all hydrocarbons
200
number of components
150
-C11H13
100
50
0
400
500
600
700
temperature (°C)

Supplement: Additional file 1 — Figure S1: Effect of temperature on the number of hydrocarbon related pyrolysis products in Botryococcus braunii. -C11H13 substructure shown as representative hydrocarbon. [file 1472-6750-10-40-S1.PPT]

## Slide 1
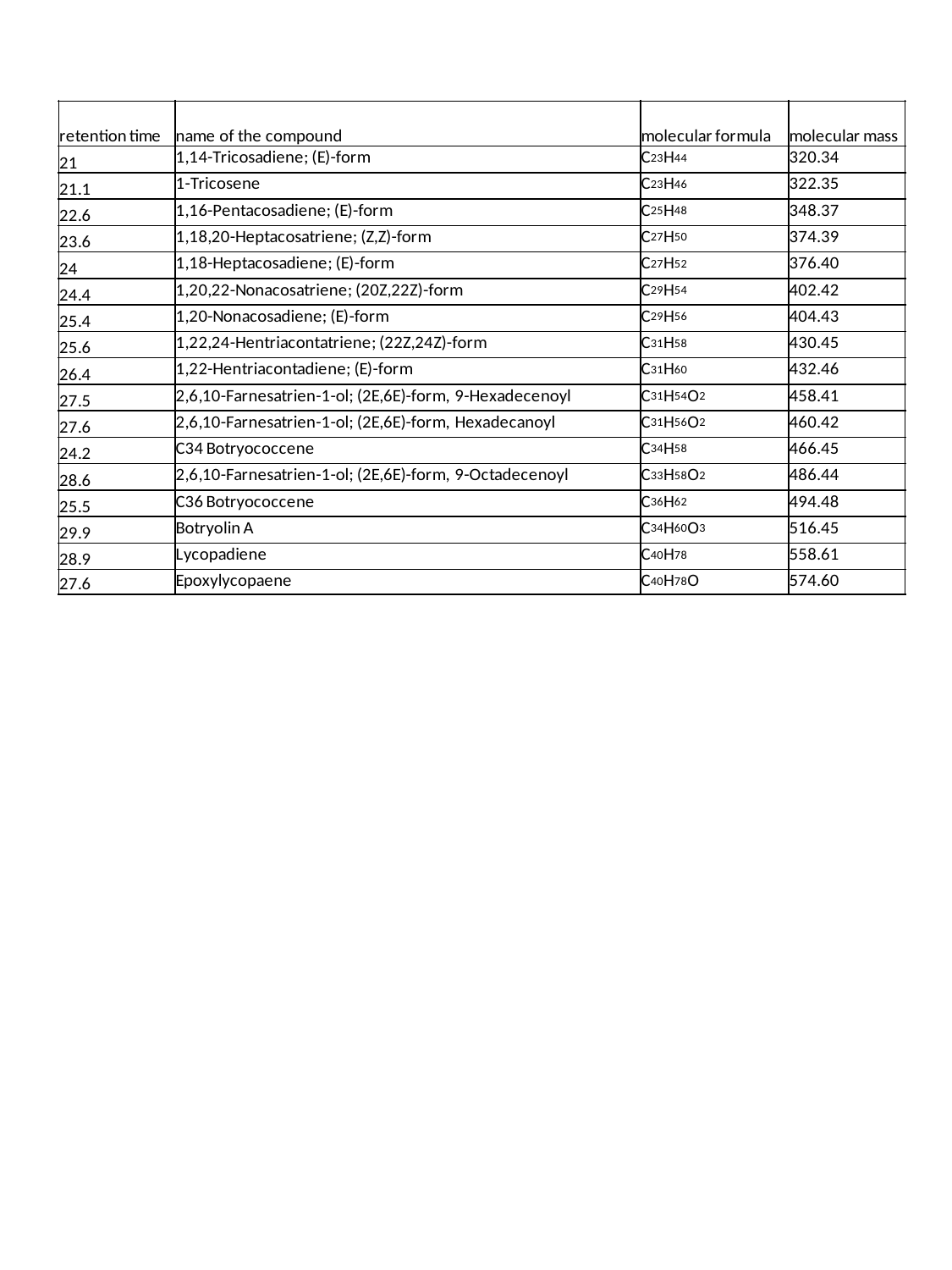

Supplement: Additional file 3 — Table S2: Compounds annotations in pyGC-MS based on matching molecular masses and fragment spectra using the Dictionary of Natural Products database. [file 1472-6750-10-40-S3.PPT]

## Slide 1
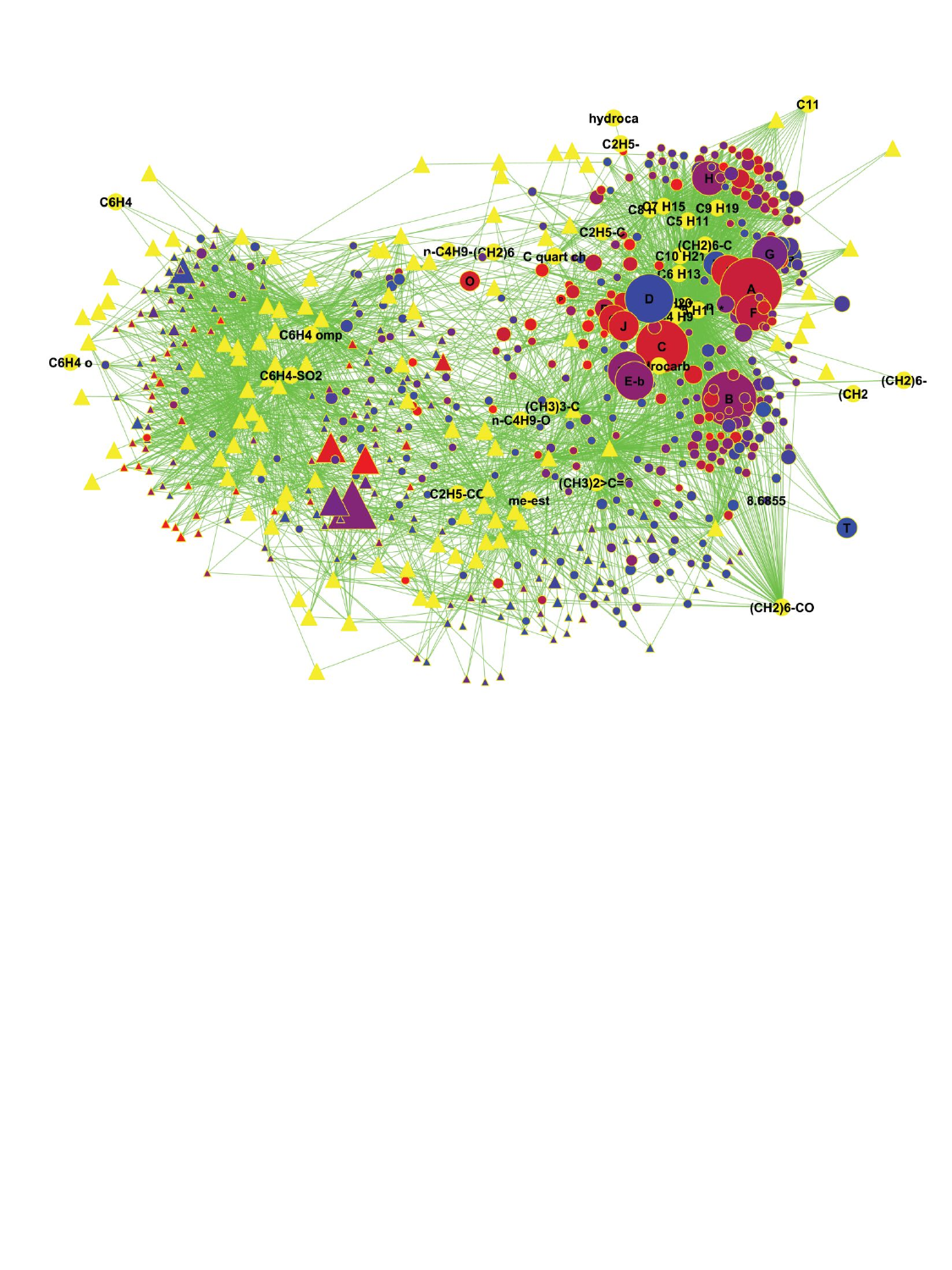

Supplement: Additional file 4 — Figure S2: Visualization of substructure annotation of using network graph in Cytoscape. Colored spheres are hydrocarbon related components. Colored triangles are non-hydrocarbon related components. Colors reflect retention time: blue = early elution; red = late elution. Yellow spheres were annotated by hydrocarbon substructure classifiers. Yellow triangles were annotated as non-hydrocarbon substructure classifiers. [file 1472-6750-10-40-S4.PPT]
